# The Effect of V_MoS3_ Point Defect on the Elastic Properties of Monolayer MoS2 with REBO Potentials

**DOI:** 10.1186/s11671-016-1377-x

**Published:** 2016-03-22

**Authors:** Minglin Li, Yaling Wan, Liping Tu, Yingchao Yang, Jun Lou

**Affiliations:** School of Mechanical Engineering and Automation, Fuzhou University, Fuzhou, 350116 China; Department of Materials Science and NanoEngineering, Rice University, Houston, TX 77005 USA

**Keywords:** Molecular dynamics simulation, Point defects, Molybdenum disulfide, Young’s modulus, REBO potential

## Abstract

Structural defects in monolayer molybdenum disulfide (MoS2) have significant influence on the electric, optical, thermal, chemical, and mechanical properties of the material. Among all the types of structural defects of the chemical vapor phase-grown monolayer MoS2, the V_MoS3_ point defect (a vacancy complex of Mo and three nearby S atoms) is another type of defect preferentially generated by the extended electron irradiation. Here, using the classical molecular dynamics simulation with reactive empirical bond-order (REBO) potential, we first investigate the effect of V_MoS3_ point defects on the elastic properties of monolayer MoS2 sheets. Under the constrained uniaxial tensile test, the elastic properties of monolayer MoS2 sheets containing V_MoS3_ vacancies with defect fraction varying from 0.01 to 0.1 are obtained based on the plane anisotropic constitutive relations of the material. It is found that the increase of V_MoS3_ vacancy concentration leads to the noticeable decrease in the elastic modulus but has a slight effect on Poisson’s ratio. The maximum decrease of the elastic modulus is up to 25 %. Increasing the ambient temperature from 10 K to 500 K has trivial influences on the elastic modulus and Poisson’s ratio for the monolayer MoS2 without defect and with 5 % V_MoS3_ vacancies. However, an anomalous parabolic relationship between the elastic modulus and the temperature is found in the monolayer MoS2 containing 0.1 % V_MoS3_ vacancy, bringing a crucial and fundamental issue to the application of monolayer MoS2 with defects.

## Background

The monolayer molybdenum disulfide (MoS2) is a graphene-like crystal with quasi-two-dimensional (2D) honeycomb lattice, consisting of a monatomic Mo-layer sandwiched between two monatomic S-layers. The pristine monolayer MoS2 holds many remarkable physical and chemical properties for its intrinsic direct bandgap of 1.8 eV [1] and high elastic modulus of ~0.2 TPa, which strongly promises for burgeoning 2D nanodevices, including transistor [2], field-effect transistor [3], phototransistors [4], nanomechanical resonator [5], and photodetector [6]. However, the structural defects can be commonly observed [7, 8] or deliberately introduced [9] in the monolayer MoS2, which have significant influence on its electrical conductivity [10], electrical contacts [11], band-to-band tunneling [12], catalytic [13], photoluminescence [14], magnetism [15], and thermal conductivity [16].

Structural defects, nine types of point defects (including vacancies and antisite defects), have been recently defined and characterized via atomic resolution imaging and first-principle calculation [7, 8]. The monosulfur vacancy (V_S_) is the most common point defect, frequently observed in experiments for its lowest formation energy (1.1 eV) [7]. So far, there are few documents concerning its impacts on the mechanical properties [17, 18], which can be momentous in MoS2 engineering applications. Dang and Spearot [17] conducted molecular dynamics (MD) nanoindentation simulations to investigate the V_S_ effect on the mechanical behavior of monolayer MoS2. They revealed that the V_S_ defects weaken the breaking force and induce displacive phase transformations under indentation. Gan and Zhao [18] performed first-principle calculations to show that the chirality effect on the mechanical properties of monolayer MoS2 becomes more and more significant with the increasing of strain, regardless of vacancies. Besides V_S_, V_MoS3_ (a vacancy complex of Mo and three nearby S atoms) is another type of defect preferentially generated by the extended electron irradiation [7]. However, there is still a lack of reports on the V_MoS3_ effect on the mechanical properties of monolayer MoS2.

Hence, in this letter, the mechanical properties of monolayer MoS2 containing V_MoS3_ (V-MoS2) with defect fraction from 0.01 to 0.1 are first investigated under the constrained uniaxial tensile test (CUATT) using MD simulation with reactive empirical bond-order (REBO) potential [19–21]. The REBO interatomic potential has been recently utilized to calculate the breaking force of monolayer MoS2 with V_S_ defects [17] and has been demonstrated to be more effective in simulating the elastic behavior of monolayer MoS2 [22] than other interatomic potentials such as consistent valence force field (CVFF) and Stillinger-Weber (SW), under a small deformation (tensile strain *ε* < 5 %). Under the CUATT, the elastic properties of monolayer MoS2 sheets containing V_MoS3_ vacancies with defect fraction varying from 0.01 to 0.1 are obtained based on the plane anisotropic constitutive relations of the material. From our simulation results, it is found that the increase of V_MoS3_ vacancy concentration leads to the noticeable decrease in the elastic modulus but has a slight effect on Poisson’s ratio. The maximum decrease of the elastic modulus is up to 25 %. Increasing the ambient temperature from 10 K to 500 K has trivial influence on the elastic modulus and Poisson’s ratio for the monolayer MoS2 without defect and with 5 % V_MoS3_ vacancies. However, an anomalous parabolic relationship between the elastic modulus and the temperature is found in the monolayer MoS2 containing 0.1 % VMoS3 vacancy, which is in conflict with the previous work using the SW potential [23] and bringing a crucial and fundamental issue to the application of monolayer MoS2 with defects.

## Methods

**Fig. 1:**
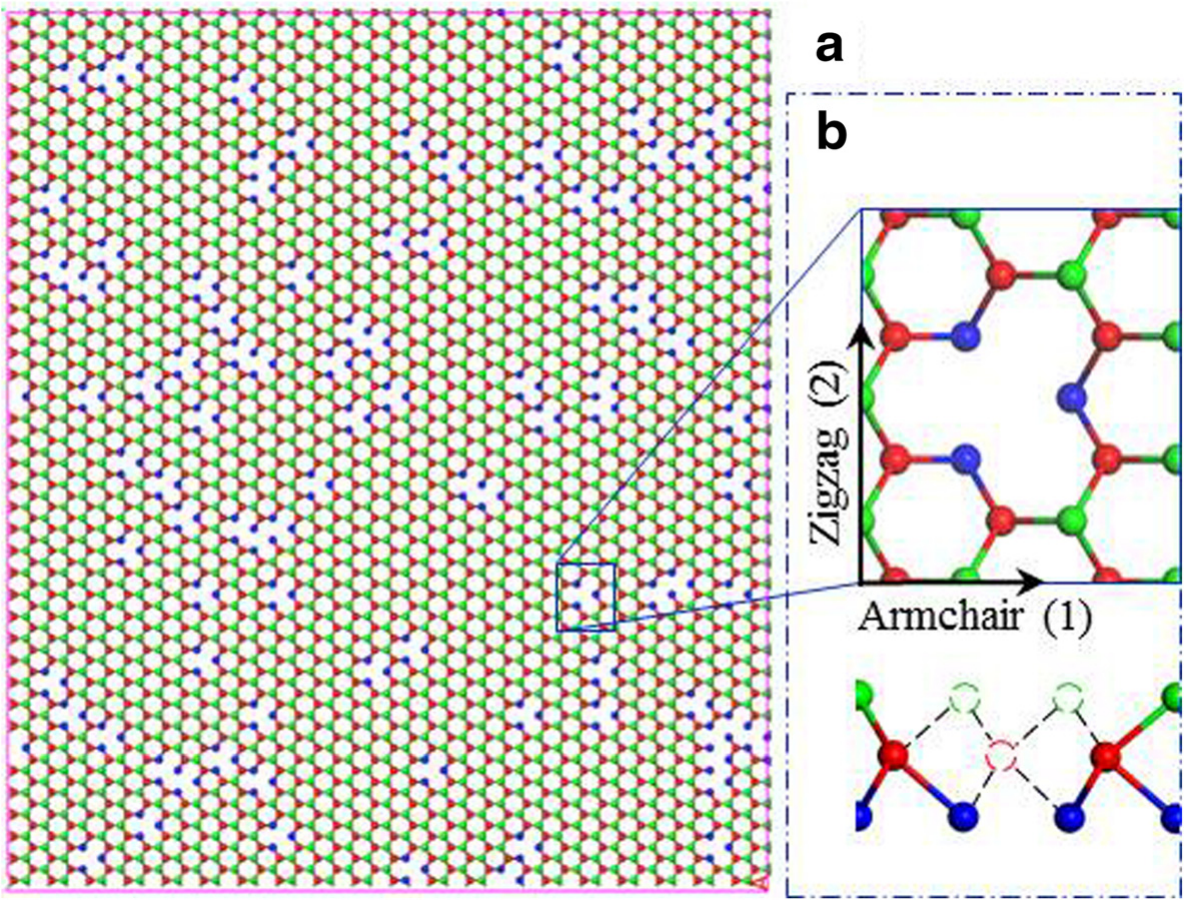
(**a**) the model of monolayer V-MoS2 sheet with 0.08 defect ratio. *Red*, *green*, and *blue balls* represent Mo, top-layer S, and bottom-layer S, respectively. (**b**) the top view of a V_MoS3_ vacancy and the direction of armchair 1 and zigzag 2 (top), the front view of a V_MoS3_ vacancy with missing dashed atoms (bottom)

The LAMMPs [24] package is utilized to perform the MD simulations. A 2D periodic boundary condition is applied to the basal plane of MoS2 in order to eliminate the boundary effect and reproduce the inherent properties of monolayer MoS2 crystals. The height of the simulation box, normal to the basal plane, is set to far larger than the thickness of monolayer MoS2 (0.65 nm) [2] and is fixed during the simulation. To construct the V_MoS3_ vacancies, randomly specified Mo atoms are first deleted and the nearby three S atoms in the bottom S-layer are then carefully removed, especially the S atoms close to the periodic boundary. Increasing the defect fraction from 0.01 to 0.1, two or more neighboring Mo atoms are apt to be deleted together to form a large hole. And some irrational S atoms, without bonding to any Mo atoms, will occur in the upper S-layer and need to be definitely avoided. Figure 1 shows one of monolayer V-MoS2 sheets (10.97 × 12.67 nm^2^) with defect fraction of 0.08.

Before performing the uniaxial tensile test using MD simulations, conjugate gradient energy minimizations are used to relax the orthorhombic simulation box as well as the atomic position of V-MoS2 sheets, to obtain the common initial configuration and the intrinsic elastic constants at absolute zero temperature (0 K). It is recently reported that the atomic relaxation has significant effects on the prediction of graphene’s elastic properties [25]. Accordingly, during the CUATT, the simulation cell length in the in-plane direction perpendicular to the applied strain keeps constant to exactly obtain the elastic constants of *C*_*ij*_ (*i*, *j* = 1, 2 for the armchair and the zigzag direction, respectively, as shown in Fig. 1b). Considering the monolayer V-MoS2 as a 2D anisotropic material, we apply the 2D orthorhombic constitutive equation [26] to display the stress-strain relationship with the chirality effect, ignoring the shear stress and strain, given as

1 $$ {\sigma}_i={C}_{ij}{\varepsilon}_j $$

Herein, *C*_*ij*_ can be expressed empirically in terms of engineering constants, modulus of elasticity *E*_*i*_ and Poisson’s ratios *ν*_*ij*_ (=−*ε*_*j*_/*ε*_*i*_), as

2 $$ {C_{ij}}_{\left(i=j\right)}={E}_i/\left(1-{\nu}_{12}{\nu}_{21}\right) $$

and

3 $$ {C_{ij}}_{\left(i\ne j\right)} = {C_{ji}}_{\left(i\ne j\right)}={\nu}_{ij}{E}_j/\left(1-{\nu}_{12}{\nu}_{21}\right) $$

Specially, *C*_11_ = *C*_22_ implies the 2D isotropic material. Furthermore, the engineering constants *E*_*i*_ and *v*_*ij*_ will be derived from the elastic constants of *C*_*ij*_ after the CUATT. The intrinsic elastic constants of *C*_*ij*_ are extracted from the slope of a perfect linear range of stress-strain curves. A strain increment of ∆*ε* = 5 × 10^−5^ is used among energy minimizations and following MD simulations [25].

## Results and Discussion

**Fig. 2:**
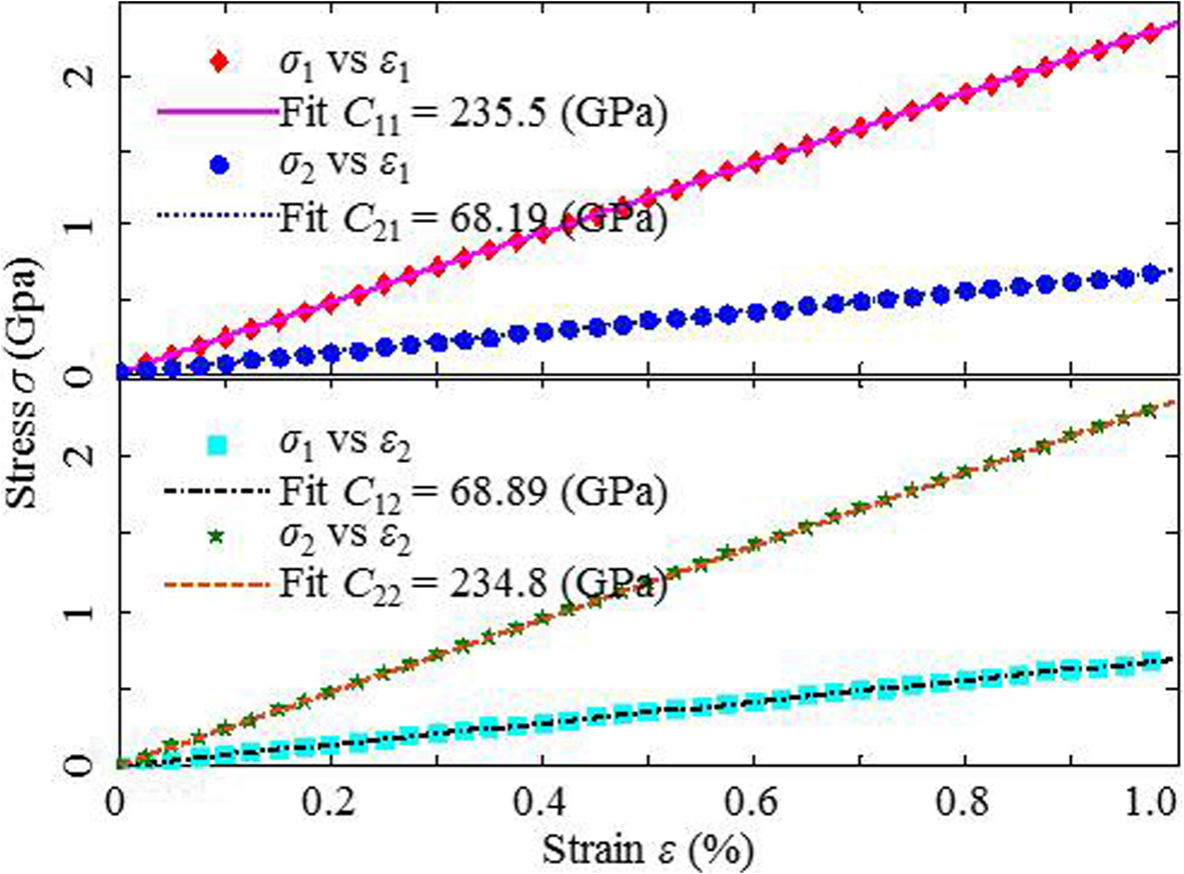
Stress-strain curves of the defect-free MoS2 sheet obtained from the armchair (*top*) and zigzag (*bottom*) loading

Figure 2 shows the stress-strain curves of the defect-free MoS2 sheet (before creating the V_MoS3_ vacancies) subjected to the CUATT and the elastic constants obtained from the slope of perfect linear curves. According to Plimpton’s study [25], the deviation of the zigzag and armchair elastic moduli (*E*_1_ = 215.76 GPa, *E*_2_ = 214.59 GPa) results in the little chirality effect. The elastic moduli of the defect-free MoS2, *E*_1_ and *E*_2_, are consistent with the experimental results of Bertolazzi etal. (270 GPa) [27] and Cooper et al. (200 GPa) [28]. The abnormal disparity between *C*_12_ and *C*_21_ is presumably due to the computational error. This is because of *C*_12_ supposed to be equal to *C*_21_, in which the stiffness matrix of materials is symmetric. Therefore, the mean value of *C*_12_ and *C*_21_ is henceforth used to assess the mechanical properties of MoS2, regardless of vacancies.

**Fig. 3:**
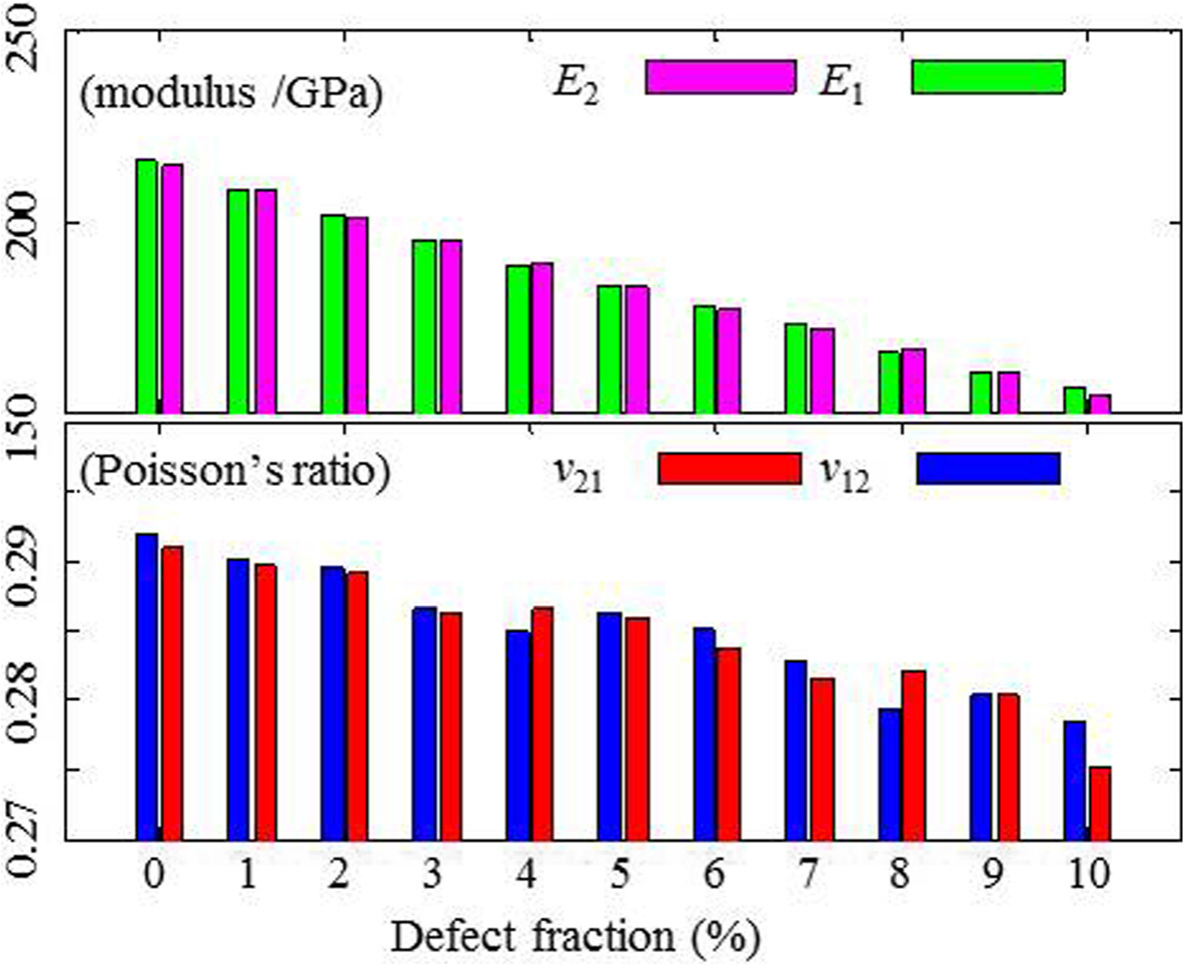
The engineering constants, elastic moduli *E* (*top*) and Poisson’s ratio *ν* (*bottom*), of V-MoS2 as a function of the defect fraction

In Fig. 3, we show the intrinsic engineering constants of V-MoS2 membranes versus the defect percentage at 0 K, compared with those of the perfect MoS2 sheet. *E*_1_ and *E*_2_ denote the elastic moduli with the armchair and zigzag directions, respectively. It is obvious that the effect of chirality on the elastic properties of V-MoS2 is negligible, regardless of the defect fraction. The V-MoS2 can be treated as isotropic 2D elastic materials due to the symmetric geometry of the V_MoS3_ vacancy. Increasing the defect fraction from 0 to 0.1, however, results in decreasing the elastic modulus *E* and Poisson’s ratio *ν*, with approximately linear relaxations. The value of elastic modulus drops obviously faster than that of Poisson’s ratio. The maximum reduction of elastic moduli is 25 %, larger than 5 % of Poisson’s ratio, which means that the impact of defect fraction on the elastic modulus is found to be more significant.

The thermal dependence of elastic properties of V-MoS2 is subsequently investigated with MD simulations, with a given defect fraction of 0.05 and under the temperature varying from 10 K to 500 K. Before the CUATT, the simulation box is relaxed for 20 ps with the NPT ensemble to bring the system to the desired temperature and pressure condition (0.1 bar). After the relaxation, the ensemble is switched to NVT, and the strain increment is applied via scaling the box length in the specified direction (zigzag for instance) and fixing the other orthogonal directions, to carry out the CUATT. The positions of system atoms are not remapped to the new box when the box is stretched, in order to keep the tensile stress consistent. In all MD simulations, the equations of motion are integrated by means of standard velocity - Verlet method with a 1-fs time step. The temperature and pressure conditions are controlled using the original Nose-Hoover thermostat and barostat.

**Fig. 4:**
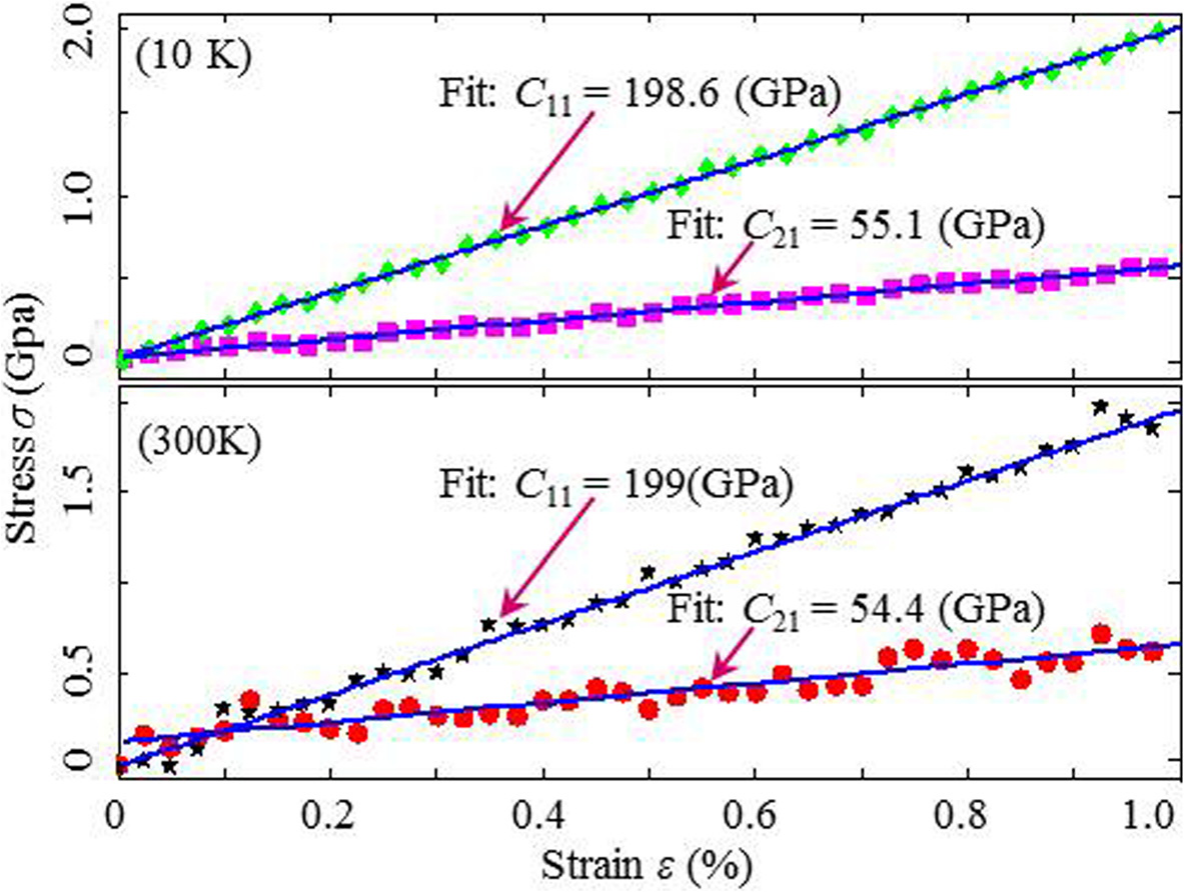
Stress-strain curves of V-MoS2 with 0.05 defect fraction, obtained from the armchair loading at 10 K (*top*) and 300 K (*bottom*) ambient temperatures

**Fig. 5:**
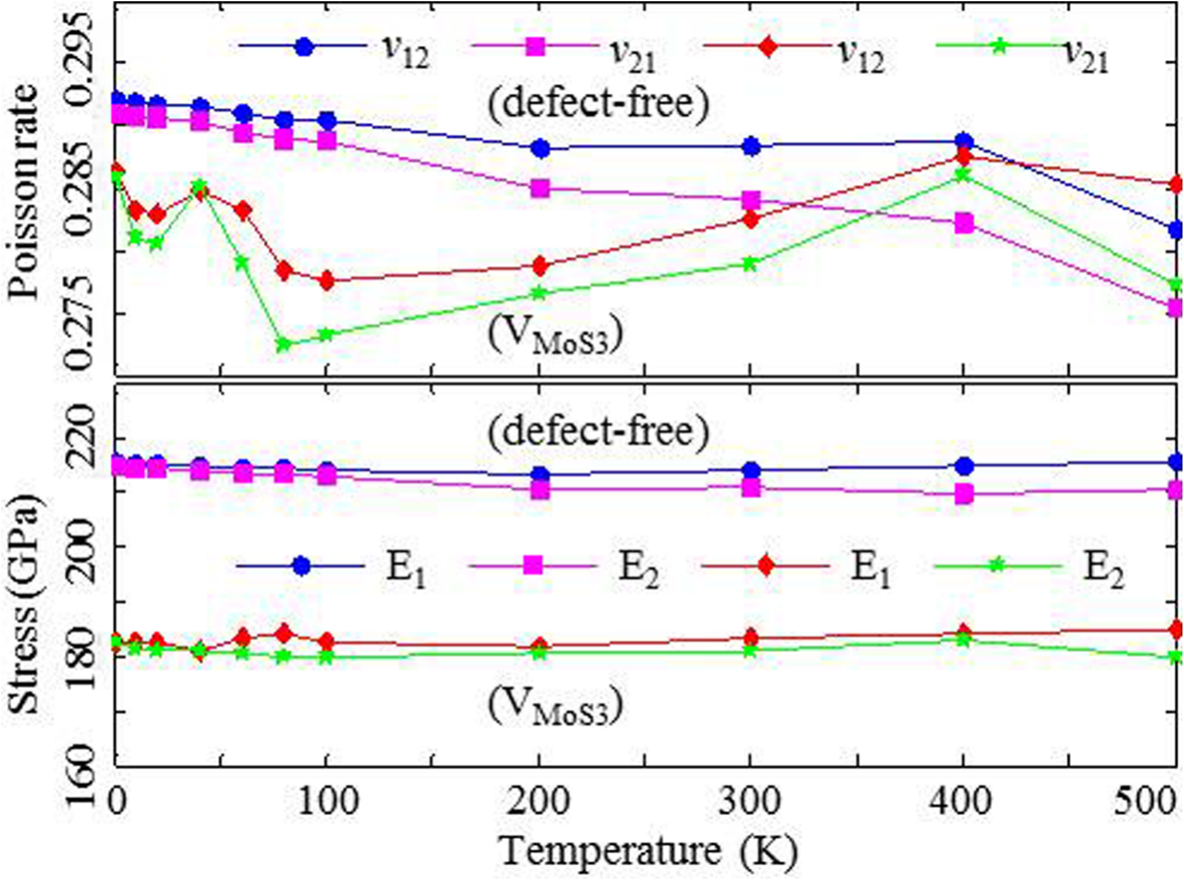
The elastic constants of the defect-free MoS2 (*circles* and *squares*) and V-MoS2 with 0.05 defect fraction (*diamonds* and *stars*) sheets versus the ambient temperature

In Fig. 4, we show the stress-strain curves of V-MoS2 with defect fraction of 0.05 under the armchair CUATT at 10 K and 300 K ambient conditions. The elastic constants of *C*_11_ and *C*_21_ at different temperatures are obtained by fitting the curves. The quality of the fit of the linear elastic model is expressed by the coefficient of determination *R*_s_. At 10 K, the value of *R*_s_ is higher than 0.99, which means that the linear model could explain 99 % of the total variability within the range of values studied. However, the fluctuation at higher temperature reduces the value of *R*_s_ down to 0.88 or even down to 0.78. Therefore, the divergence of elastic constants among the temperatures varying from 10 K to 500 K can be neglected, which demonstrates in Fig. 5 that temperatures lower than 500 K have basically little effect on the elastic properties (less than 5 %), including the elastic modulus and Poisson’s ratio, regardless of vacancies.

As shown in Fig. 5, Poisson’s ratio *ν*_12_ (circles) and *ν*_21_ (squares) of the defect-free MoS2 sheets slightly decrease as the temperature increases, excluding the data of *ν*_12_ at 300 K and 400 K. However, Poisson’s ratio *ν*_12_(diamonds) and *ν*_21_ (stars) of V-MoS2 sheets slightly fluctuate as the temperature increases, in which the maximum amplitude does not exceed 2 %. The fluctuation can be attributed to the vacancies, which allow the ambient atoms to vibrate violently. As for Young’s modulus, the defect-free MoS2 sheet and the V-MoS2 sheet both show little dependence on the system temperature.

The temperature dependence of the defect-free MoS2 sheet obtained from our simulations with REBO potential is entirely contrary to the work of Zhao etal. [23] using the SW potential, in which Young’s modulus of perfect monolayer MoS2 obviously decreases with increasing the ambient temperature from 4.2 K to 500 K. They obtained the maximum reduction of Young’s modulus more than 30 %. However, the temperature dependence of the defect-free MoS2 with REBO potential in this paper is comparable to that of graphene [29], in which the maximum reduction of Young’s modulus is about 5 % when the system temperature increases from 300 K to 700 K. We believe that such result distinction is mainly derived from the adoption of different interatomic potentials and the processing procedure, as the co-worker of Zhao published another totally different result [30], in which Young’s modulus of perfect MoS2 is independent to the temperature range from 0 K to 300 K.

**Fig. 6:**
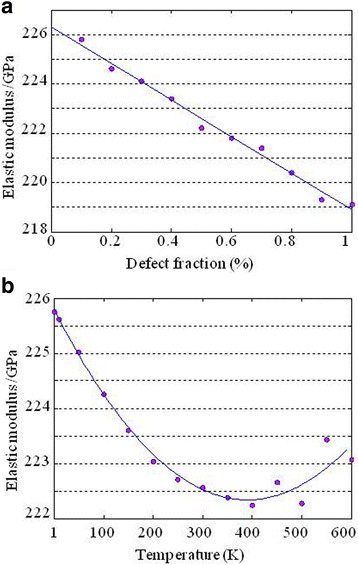
The elastic modulus varying with **a** defect fractions ranging from 0.1 to 1 % and **b** temperatures from 1 K to 600 K

Recent experimental and theoretical nanoindentation studies have revealed that the low concentration of monovacancy leads to an anomalous remarkable stiffening effect on the graphene membrane [31–33]. Further simulation results [31] indicated that other types of point defects, such as divacancy, 555–777, and Stone-Wales defects, did not augment the in-plane stiffness of graphene but led to the ordinary degradation. As for the monolayer MoS2, which consists of stacks of S-Mo-S sandwiches, does the low fraction of V_MoS3_ vacancies lead to remarkable stiffening effect in a similar way? Considering that its crystal lattice and structural defects are distinct to those of graphene, a crucial and fundamental issue about the effect of low defect concentration on the mechanical properties of low dimensional nanomaterials is now brought up to scientists. Herein, we make a preliminary investigation on this issue. Constructing monolayer V-MoS3 sheet with defect fractions from 0.1 % to 1 %, we obtained the elastic modulus varying with defect fractions and temperatures from 1 K to 600 K. Surprisingly, the elastic modulus of monolayer MoS2 decreases monotonously as the defect fraction of V_MoS3_ increases, as shown in Fig. 6a. Moreover, the parabolic relationship between the elastic modulus of monolayer MoS2 containing 0.1 % V_MoS3_ vacancy and the temperature shows the anomalous temperature dependency of the elastic modulus of monolayer MoS2 with low concentration of V_MoS3_, as shown in Fig. 6b. However, note that there is a discrepancy in the testing method of mechanical properties between our results and those of literatures [31, 32]. We used uniaxial traction simulations instead of nanoindentation simulations. Hence, more comprehensive investigations combining the nanoindentation and uniaxial traction simulations are needed and now going on in our group, which will be submitted in the other manuscript.

## Conclusions

In conclusion, we first investigate the mechanical properties and the thermal dependence of monolayer MoS2 containing V_MoS3_ vacancies with defect fraction varying from 0.01 to 0.1 under the constrained uniaxial tensile test using MD simulation with REBO potential. Our simulation results show that the V_MoS3_ vacancy concentration has noticeable influence on the elastic modulus but has a slight effect on Poisson’s ratio. Increasing the ambient temperature from 10 K to 500 K has trivial influence on the elastic modulus and Poisson’s ratio for the monolayer MoS2 without defect and with 5 % V_MoS3_ vacancies. However, an anomalous parabolic relationship between the elastic modulus and the temperature is found in the monolayer MoS2 containing 0.1 % VMoS3 vacancy and bringing a crucial and fundamental issue to the application of monolayer MoS2 with defects.
